# Angiopoietin-1 Treatment Reduces Inflammation but Does Not Prevent Ventilator-Induced Lung Injury

**DOI:** 10.1371/journal.pone.0015653

**Published:** 2010-12-14

**Authors:** Maria A. Hegeman, Marije P. Hennus, Matijs van Meurs, Pieter M. Cobelens, Annemieke Kavelaars, Nicolaas J. Jansen, Marcus J. Schultz, Adrianus J. van Vught, Grietje Molema, Cobi J. Heijnen

**Affiliations:** 1 Laboratory of Neuroimmunology and Developmental Origins of Disease, University Medical Center Utrecht, Utrecht, The Netherlands; 2 Department of Pediatric Intensive Care, University Medical Center Utrecht, Utrecht, The Netherlands; 3 Department of Critical Care, University Medical Center Groningen, University of Groningen, Groningen, The Netherlands; 4 Medical Biology Section, Department of Pathology and Medical Biology, University Medical Center Groningen, University of Groningen, Groningen, The Netherlands; 5 Department of Intensive Care Medicine, University Medical Center Utrecht, Utrecht, The Netherlands; 6 Laboratory of Experimental Intensive Care and Anesthesiology, Department of Intensive Care Medicine, Academic Medical Center, Amsterdam, The Netherlands; University of Alabama-Birmingham, United States of America

## Abstract

**Background:**

Loss of integrity of the epithelial and endothelial barriers is thought to be a prominent feature of ventilator-induced lung injury (VILI). Based on its function in vascular integrity, we hypothesize that the angiopoietin (Ang)-Tie2 system plays a role in the development of VILI. The present study was designed to examine the effects of mechanical ventilation on the Ang-Tie2 system in lung tissue. Moreover, we evaluated whether treatment with Ang-1, a Tie2 receptor agonist, protects against inflammation, vascular leakage and impaired gas exchange induced by mechanical ventilation.

**Methods:**

Mice were anesthetized, tracheotomized and mechanically ventilated for 5 hours with either an inspiratory pressure of 10 cmH_2_O (‘low’ tidal volume ∼7.5 ml/kg; LV_T_) or 18 cmH_2_O (‘high’ tidal volume ∼15 ml/kg; HV_T_). At initiation of HV_T_-ventilation, recombinant human Ang-1 was intravenously administered (1 or 4 µg per animal). Non-ventilated mice served as controls.

**Results:**

HV_T_-ventilation influenced the Ang-Tie2 system in lungs of healthy mice since Ang-1, Ang-2 and Tie2 mRNA were decreased. Treatment with Ang-1 increased Akt-phosphorylation indicating Tie2 signaling. Ang-1 treatment reduced infiltration of granulocytes and expression of keratinocyte-derived chemokine (KC), macrophage inflammatory protein (MIP)-2, monocyte chemotactic protein (MCP)-1 and interleukin (IL)-1β caused by HV_T_-ventilation. Importantly, Ang-1 treatment did not prevent vascular leakage and impaired gas exchange in HV_T_-ventilated mice despite inhibition of inflammation, vascular endothelial growth factor (VEGF) and Ang-2 expression.

**Conclusions:**

Ang-1 treatment downregulates pulmonary inflammation, VEGF and Ang-2 expression but does not protect against vascular leakage and impaired gas exchange induced by HV_T_-ventilation.

## Introduction

Mechanical ventilation is an important life-saving procedure. However, the procedure itself may induce or aggravate damage to lung tissue, so-called ventilator-induced lung injury (VILI) [1], [2]. VILI is characterized by inflammation, enhanced alveolar-capillary membrane permeability, accumulation of protein-rich pulmonary edema and ultimately impaired gas exchange [3]. Various animal models have been used to obtain further insight into the mechanisms underlying VILI. Already in the 1980s, investigators showed that mechanical ventilation and the subsequent mechanical (over)stretch of lung tissue induces damage to the epithelial-endothelial barrier leading to impaired oxygenation [4]–[6]. In addition, it has been described that the mechanical forces associated with mechanical ventilation provoke an inflammatory response in the lung (biotrauma) [7], [8].

Loss of integrity of epithelial and endothelial cell monolayers has been suggested to play an important role in the ventilator-induced disruption of the alveolar-capillary barrier [9]. One of the crucial systems regulating vascular cell integrity is the angiopoietin (Ang)-Tie2 system [10]. Clarifying the role of the Ang-Tie2 system in the development of lung injury has therefore become a topic of great interest [11]. However, to date little is known about the interaction of mechanical (over)stretch with the Ang-Tie2 system. It has been recognized that Ang-1 serves as a Tie2 receptor agonist by phosphorylating Tie2 on tyrosine residues. Ang-1−mediated Tie2 signaling is required to maintain cellular integrity and quiescence of the endothelial barrier [10]. The antagonist Ang-2 is known to downregulate Tie2 signaling, thereby preparing vascular endothelial cells for enhanced responsiveness to factors that cause destabilization of the endothelial barrier [12]. However, there is also conflicting evidence that Ang-2 may cause Tie2 activation in stressed endothelial cells [13].

In a murine model of endotoxin-induced acute lung injury (ALI), Karmpaliotis et al. described that vascular permeability and pulmonary edema were accompanied by enhanced vascular endothelial growth factor (VEGF) and reduced Ang-1 levels in lung tissue [14]. The same authors proposed that changes in the balance between VEGF (pro-leakage) and Ang-1 (anti-leakage) might contribute to the pathophysiology of ALI. Protective effects of Ang-1 treatment have been shown before in experimental models of endotoxin-induced ALI [15]–[18]. Mei et al. demonstrated that treatment with Ang-1 attenuated vascular leakage, granulocyte infiltration and pro-inflammatory cytokine expression in lungs of endotoxin-exposed mice [17]. Consequently, the Ang-Tie2 system has been proposed as a possible therapeutic target in pulmonary diseases like ALI and its most severe form, the acute respiratory distress syndrome (ARDS) [11], [19].

Vascular leakage and pulmonary inflammation are important features of VILI. Therefore, we hypothesized that Ang-1−Tie2 signaling plays a (protective) role in the development of VILI. In an attempt to better reflect the human setting, we applied a relatively mild model of VILI using clinically relevant ventilator settings thereby preventing shock, metabolic acidosis and substantial damage to lung architecture [20]. The aim of present study was to investigate the influence of mechanical ventilation on the Ang-Tie2 system in lungs of healthy adult mice. Furthermore, we examined whether treatment with Ang-1, a Tie2 receptor agonist, would protect ventilated mice against important hallmarks of VILI such as inflammation, vascular leakage and impaired gas exchange.

## Methods

### Animals

Experiments were performed in accordance with international guide lines and approved by the animal care and use committees of the University Medical Center Utrecht and the Academic Medical Center Amsterdam (approval IDs: 2007.II.02.066 and DAA101607). Adult male C57Bl6 mice (n = 145; Charles River, Maastricht, the Netherlands), weighing 20 to 24 grams, were randomly assigned to different experimental groups.

Mice (n = 114) were ventilated for 5 hours as described previously [20]; pressure-controlled, fractional inspired oxygen concentration (FiO_2_) of 0.5, inspiration-to-expiration ratio of 1:1 and positive end-expiratory pressure of 2 cmH_2_O. Six mice were ventilated simultaneously with either an inspiratory pressure of 10 cmH_2_O (resulting in ‘low’ tidal volume (V_T_) ∼7.5 ml/kg; LV_T_) or 18 cmH_2_O (resulting in ‘high’ V_T_ ∼15 ml/kg; HV_T_). Respiratory rate was set at 100 and 50 breaths/min, respectively. Body temperature was kept constant between 36.5 and 37.5°C. Non-ventilated mice (n = 31) served as controls (non-ventilated controls, NVC).

### Ang-1 treatment

At initiation of HV_T_-ventilation, recombinant human Ang-1 (carrier free; R&D systems, Minneapolis, MN) was intravenously administered (either 1 or 4 µg per animal). The dose of 1 µg has been shown to be efficient in attenuating lung inflammation and injury [21]. Control HV_T_-ventilated mice received the same volume of sterile saline (vehicle) intravenously.

### Hemodynamics and blood gas analysis

After 0, 2.5 and 5 hours, systolic blood pressure and heart rate were non-invasively monitored using a tail-cuff system (ADInstruments, Spenbach, Germany). After 5 hours, arterial blood was taken from the carotid artery for blood gas analysis (Rapidlab 865; Bayer, Mijdrecht, the Netherlands).

### Bronchoalveolar lavage

The right lung was lavaged by instilling 3×0.5 ml sterile saline. Differential counts were done on cytospin preparations stained with Giemsa (Diff-Quick; Dade Behring AG, Düdingen, Switzerland). Cell-free supernatant was used to measure total protein (BCA protein-assay; Pierce Biotechnology, Rockford, IL) with BSA as standard.

### Wet-to-dry ratio

The left lung was weighed, dried for 3 days (65°C) and weighed again.

### Histology and immunohistochemistry

The left lung was filled with Tissue-Tek® (Sakura Finetek, Zoeterwoude, the Netherlands), snap frozen and cut to 5 µm cryosections using a cryostat. To assess pulmonary histopathology, sections were stained with hematoxylin-eosin (H&E; Klinipath, Duiven, the Netherlands). To assess Tie2 localization, sections were stained with fluorescent antibody recognizing Tie2 (Tek4; eBioscience, San Diego, CA) or isotype control antibody (IgG1-biotin).

### Tissue homogenate preparation

Lung tissue of separate animals were pulverized using a liquid nitrogen-cooled mortar and pestle, and divided in several fractions allowing us to perform multiple analyses (as described below).

### Real-time RT-PCR analysis

PCR was performed as described previously [22]. Primer sequences: Ang-1, forward CTACCAACAACAACAgCATCC, reverse CTCCCTTTAgCAAAACACCTTC; Ang-2, forward CTgTgCggAAATCTTCAAgTC, reverse TgCCATCTTCTCggTgTT; Tie2, forward gTgTAgTggACCAgAAgg, reverse CTTgAgAgCAgAggCATC. Data were normalized for expression of internal controls, i.e. the average value of β-actin and glyceraldehyde 3-phosphate dehydrogenase (GAPdH).

### Western blotting

Western blotting was performed as described previously [23]. Antibodies recognized phospho (p)-Akt (Ser473; Cell Signaling, Danvers, MA), Ang-1 or Ang-2 (both Alpha Diagnostic, San Antonio, TX). To control for equal loading, membranes were stripped when necessary and reprobed with an antibody recognizing total Akt (Akt1/PKBα; Sigma-Aldrich, Steinheim, Germany). Total Akt was chosen as a loading control since mechanical ventilation influenced expression levels of β-actin in our experimental model of VILI.

### ELISA

Tie2 protein was measured by ELISA according to manufacturer's instructions (R&D).

### Myeloperoxidase (MPO) activity

MPO activity was determined as described previously [22].

### Multiplex cytokine assay

125 µg protein was analyzed for keratinocyte-derived chemokine (KC), macrophage inflammatory protein (MIP)-2, monocyte chemotactic protein (MCP)-1, interleukin (IL)-1β, IL-6, IL-10 and VEGF by multiplex cytokine assay using a Luminex analyzer (Bio-Rad Laboratories, Hercules, CA) according to manufacturer's instructions (multiplex mouse cytokine, R&D).

### Statistical analysis

Data are expressed as mean ± SEM. Oxygenation variables (LV_T_ versus HV_T_) and Ang-1, Ang-2, Tie2 protein (NVC versus HV_T_) were analyzed by independent T-test. Other parameters were analyzed by one-way ANOVA with least significant difference (LSD) post-hoc test. P-values less than 0.05 were considered statistically significant.

## Results

### Stability of the murine model of VILI

All mice survived 5 hours of LV_T_ and HV_T_-ventilation after which they were sacrificed. Analysis of systolic blood pressure and heart rate revealed stable conditions of ventilated mice throughout the experiment (table 1).

**Table 1:** Hemodynamic characteristics over 5 hours of mechanical ventilation.

	LV_T_	HV_T_
HR _t = 0 hr_	370±11	387±17
HR _t = 2,5 hr_	382±11	336±13
HR _t = 5 hr_	403±12	351±9
BP _t = 0 hr_	104±4	97±6
BP _t = 2,5 hr_	76±6	72±4
BP _t = 5 hr_	73±5	74±5

LV_T_, HV_T_  =  mice ventilated with low or high tidal volumes; HR  =  heart rate in beats per minute, BP  =  systolic blood pressure in mmHg. Data are presented as mean ± SEM of 11 to 12 animals per group.

Table 2 illustrates that arterial oxygen tension (PaO_2_) was reduced in HV_T_-ventilated mice when compared to LV_T_-ventilated mice. Carbon dioxide tension (PaCO_2_), pH and base excess (BE) remained within the physiological range during both LV_T_ and HV_T_-ventilation.

**Table 2:** Arterial blood gas analysis after 5 hours of mechanical ventilation.

	LV_T_	HV_T_
PaO_2_	231.6±15.8	165.8±12.8*
PaCO_2_	32.4±3.4	35.1±2.5
pH	7.51±0.03	7.50±0.02
BE	2.08±0.78	3.54±0.91

LV_T_, HV_T_  =  mice ventilated with low or high tidal volumes; PaO_2_  =  partial pressure of arterial oxygen in mmHg; PaCO_2_  =  partial pressure of arterial carbon dioxide in mmHg; BE  =  base excess in mmol/l. Data are presented as mean ± SEM of 16 to 18 animals per group (*p<0.01 versus LV_T_).

### Effect of mechanical ventilation on alveolar-capillary permeability, pulmonary edema formation and gas exchange

**Figure 1:**
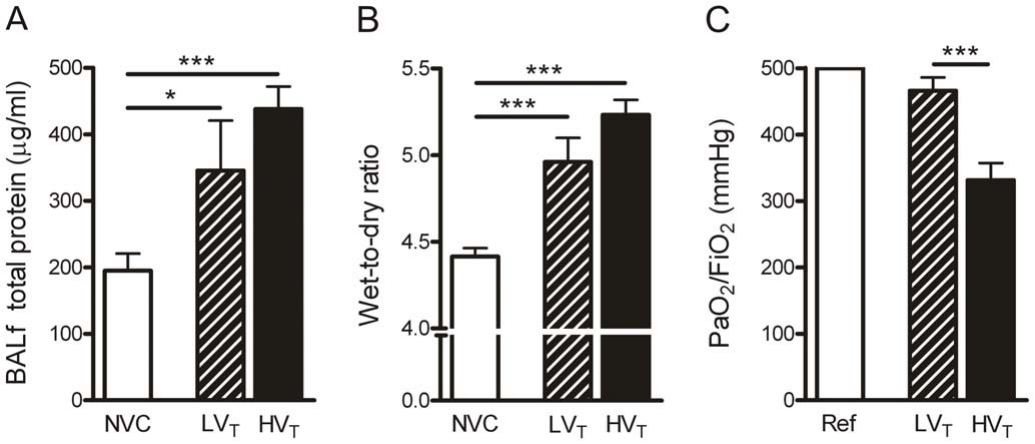
Mechanical ventilation affects alveolar-capillary permeability, pulmonary edema formation and gas exchange. A: Alveolar-capillary permeability is represented by total protein levels in bronchoalveolar lavage fluid (BALf). B: Pulmonary edema is represented by wet-to-dry ratios of lung tissue. C: Oxygenation is represented by the ratio of partial pressure arterial oxygen and fraction inspired oxygen (PaO_2_/FiO_2_). Data are expressed as mean ± SEM of 9-12 (A–B) or 18–21 (C) animals for each group (* p<0.05, *** p<0.001). Ref  =  reference bar (PaO_2_/FiO_2_ ratio for mice with non-injured lungs); NVC  =  non-ventilated controls; LV_T_, HV_T_  =  mice ventilated with low or high tidal volumes.

First, we examined whether LV_T_ and HV_T_-ventilation caused lung injury in mice without pre-existing lung injury. Total protein levels in bronchoalveolar lavage fluid (BALf), wet-to-dry ratios of pulmonary tissue and PaO_2_/FiO_2_ ratio in blood samples were analyzed to evaluate the effects of mechanical ventilation on alveolar-capillary permeability, pulmonary edema formation and gas exchange respectively. We found that both ventilation strategies markedly enhanced BALf protein levels and pulmonary wet-to-dry ratios in comparison with NVC (figures 1a and b). Furthermore, PaO_2_/FiO_2_ ratios were significantly decreased when comparing HV_T_-ventilated mice with LV_T_-ventilated mice (figure 1c).

### Effect of mechanical ventilation on the Ang-Tie2 system

**Figure 2:**
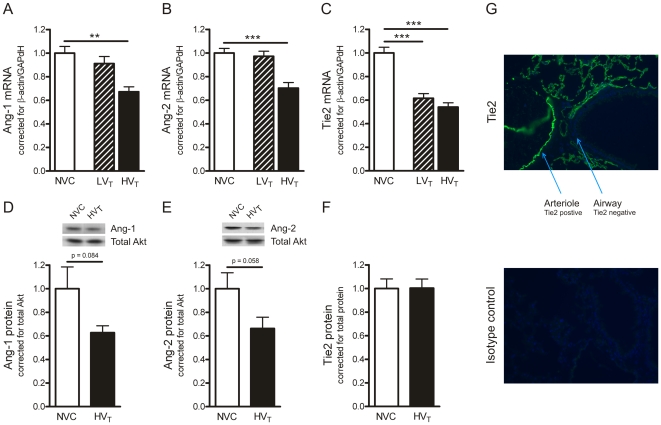
Mechanical ventilation affects the angiopoietin (Ang)-Tie2 system. A–C: In total lung homogenates, mRNA expression of Ang-1, Ang-2 and Tie2 was determined by real time RT-PCR. Levels were normalized for expression of internal controls, i.e. the average value of β-actin and glyceraldehyde 3-phosphate dehydrogenase (GAPdH). No group differences in expression levels of β-actin and GAPdH were observed. D–E: In total lung homogenates, protein expression of Ang-1 and Ang-2 was determined by Western blotting. Membranes were reprobed with antibody recognizing total Akt (Akt1/PKBα) to control for equal loading. No group differences in total Akt were observed. Inset: representative Western Blot depicting immunodetectable Ang-1 and Ang-2. F: In total lung homogenates, protein expression of Tie2 was determined by ELISA. Levels were normalized for total protein concentrations. Data are depicted relative to NVC and expressed as mean ± SEM of 6–16 animals for each group (** p<0.01, *** p<0.001). G: Lung sections of non-ventilated controls were stained with fluorescent antibody recognizing Tie2 to visualize the presence of Tie2 on pulmonary cells (isotype control was negative). Magnification ×200. NVC  =  non-ventilated controls; LV_T_, HV_T_  =  mice ventilated with low or high tidal volumes.

To assess whether 5 hours of mechanical ventilation influenced the Ang-Tie2 system, we determined Ang-1, Ang-2 and Tie2 expression in total lung homogenates. We found that only HV_T_-ventilation caused a decrease in Ang-1 and Ang-2 mRNA compared to NVC (figures 2a and b). Both ventilation strategies reduced Tie2 mRNA (figure 2c). Since ventilator-induced effects on the Ang-Tie2 system, vascular leakage and oxygenation were most pronounced after HV_T_-ventilation, we continued our investigations by focusing on this specific group.

To determine if ventilator-induced reduction of Ang-1, Ang-2 and Tie2 mRNA also resulted in a reduction of Ang-1, Ang-2 and Tie2 protein, we measured protein expression of these mediators in total lung homogenates. Decreased Ang-1 and Ang-2 protein levels were observed after 5 hours of HV_T_-ventilation, although these differences did not reach statistical significance (p = 0.084 and p = 0.058 respectively, figures 2d and e). In addition, HV_T_-ventilation did not lead to downregulation of Tie2 protein expression (figure 2f). To evaluate the localization of Tie2 in pulmonary tissue, Tie2 protein was visualized by immunofluorescent staining on lung sections of NVC. As illustrated in figure 2g, Tie2 was primarily detected in larger arterioles and alveolar-capillary membranes (isotype control was negative).

### Effect of Ang-1 treatment on phospho-Akt (p-Akt), Ang-2 and VEGF expression

**Figure 3:**
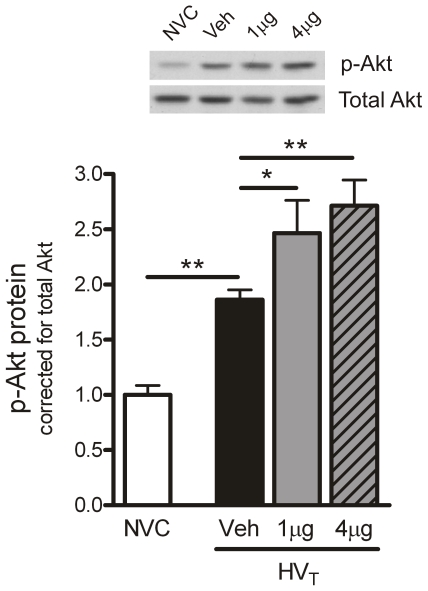
Angiopoietin (Ang)-1 treatment induces Tie2 signaling. In total lung homogenates, protein expression of p-Akt was determined by Western blotting as an indirect measure of Tie2 signaling. Membranes were stripped and reprobed with antibody recognizing total Akt (Akt1/PKBα) to control for equal loading. No group differences in total Akt were observed. Inset: representative Western blot depicting immunodetectable p-Akt. Data are depicted relative to NVC and expressed as mean ± SEM of 8–10 animals per group (* p<0.05, ** p<0.01). NVC  =  non-ventilated controls; HV_T_  =  mice ventilated with high tidal volumes; Veh, 1 µg, 4 µg  =  mice intravenously treated with either vehicle (sterile saline), Ang-1 (1 µg per animal), or Ang-1 (4 µg per animal).

Mice were treated with either sterile saline (vehicle), 1 µg Ang-1 or 4 µg Ang-1 per animal at initiation of HV_T_-ventilation and subsequently ventilated for 5 hours. No changes in hemodynamic and blood gas variables were observed after Ang-1 treatment (data not shown). We determined the level of p-Akt protein. Akt phosphorylation is known to be induced by Ang-1–mediated Tie2 signaling, and thus, increased levels of p-Akt may serve as indirect evidence of Tie2 receptor activation. HV_T_-ventilation induced phosphorylation of Akt compared to NVC and Ang-1 treatment enhanced the level of p-Akt even further (figure 3). This increase was most distinct when using a dose of 4 µg Ang-1 per animal.

**Figure 4:**
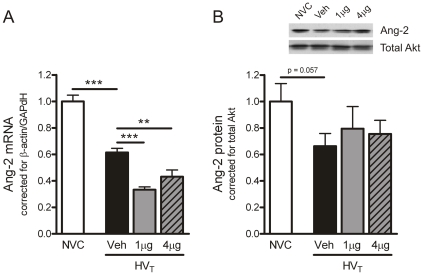
Angiopoietin (Ang)-1 treatment reduces Ang-2 mRNA expression. A: In total lung homogenates, mRNA expression of Ang-2 was determined by real time RT-PCR. Levels were normalized for expression of internal controls, i.e. the average value of β-actin and glyceraldehyde 3-phosphate dehydrogenase (GAPdH). No group differences in β-actin and GAPdH were observed. B: In total lung homogenates, protein expression of Ang-2 was determined by Western blotting. Membranes were reprobed with antibody recognizing total Akt (Akt1/PKBα) to control for equal loading. No group differences in total Akt were observed. Inset: representative Western blot depicting immunodetectable Ang-2. Data are depicted relative to NVC and expressed as mean ± SEM of 7–16 animals per group (** p<0.01, *** p<0.001). NVC  =  non-ventilated controls; HV_T_  =  mice ventilated with high tidal volumes; Veh, 1 µg, 4 µg  =  mice intravenously treated with either vehicle (sterile saline), Ang-1 (1 µg per animal), or Ang-1 (4 µg per animal).

**Figure 5:**
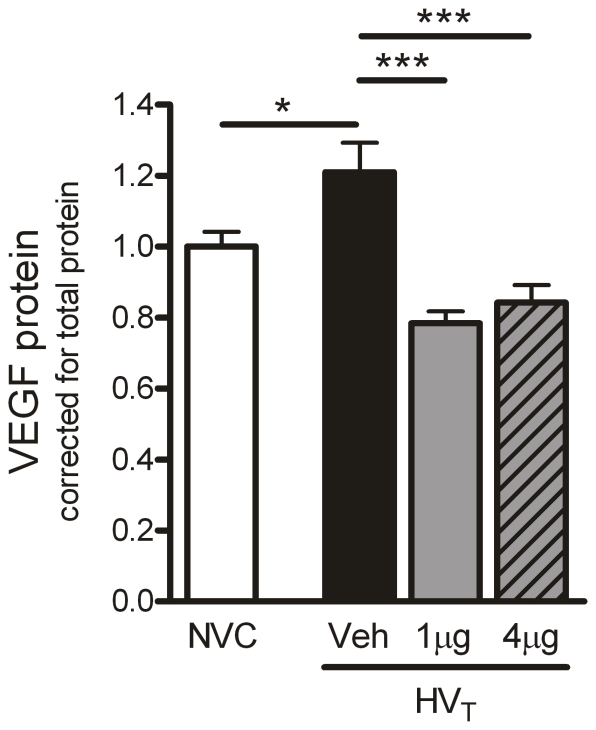
Angiopoietin (Ang)-1 treatment reduces vascular endothelial growth factor (VEGF) protein expression. In total lung homogenates, protein expression of VEGF was determined by multiplex cytokine analysis. Levels were normalized for total protein concentrations. No group differences in tissue total protein were observed. Data are depicted relative to NVC and expressed as mean ± SEM of 8–10 animals (* p<0.05, *** p<0.001). NVC  =  non-ventilated controls; HV_T_  =  mice ventilated with high tidal volumes; Veh, 1 µg, 4 µg  =  mice intravenously treated with either vehicle (sterile saline), Ang-1 (1 µg per animal), or Ang-1 (4 µg per animal).

We also studied if Ang-1 treatment altered the expression of factors promoting vascular leakage like Ang-2 and VEGF. On mRNA level, Ang-2 expression was downregulated in lungs of HV_T_-ventilated mice treated with 1 or 4 µg Ang-1 (figure 4a). At this time point, no effect of Ang-1 treatment was observed on Ang-2 protein expression (figure 4b). Compared to NVC, VEGF protein expression was increased in the vehicle-treated HV_T_-group (figure 5). Both doses of Ang-1 completely abolished the increase in VEGF protein in response to HV_T_-ventilation.

### Effect of Ang-1 treatment on ventilator-induced granulocyte infiltration in pulmonary tissue and granulocyte exudation into the alveolar space

**Figure 6:**
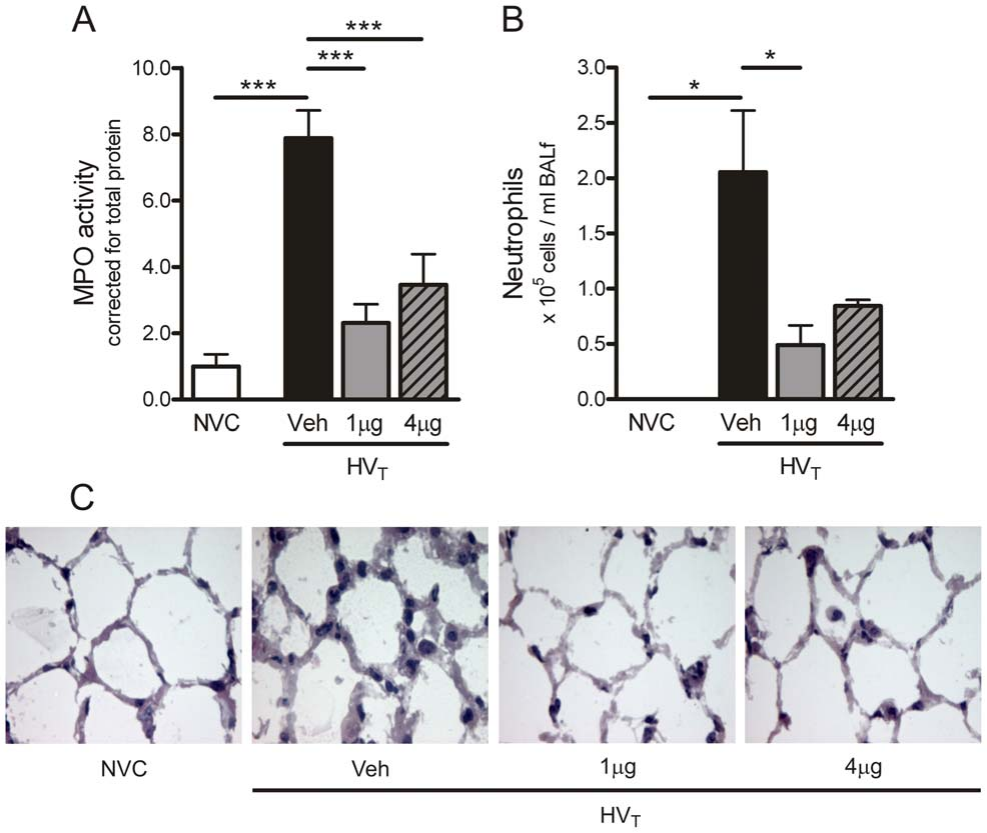
Angiopoietin (Ang)-1 treatment reduces granulocyte infiltration. A: In total lung homogenates, myeloperoxidase (MPO) activity was determined as a measure of granulocyte infiltration. Levels were normalized for total protein concentrations. No group differences in tissue total protein were observed. B: On cytospin preparations, differential cell counts were done to determine neutrophil exudation in the alveolar space. Data are depicted relative to NVC and expressed as mean ± SEM of 5–16 animals per group (* p<0.05, *** p<0.001). C: Lung sections were stained with hematoxylin-eosin (H&E) to analyze the presence of granulocytes in pulmonary tissue. Magnification ×500. NVC  =  non-ventilated controls; HV_T_  =  mice ventilated with high tidal volumes; Veh, 1 µg, 4 µg  =  mice intravenously treated with either vehicle (sterile saline), Ang-1 (1 µg per animal), or Ang-1 (4 µg per animal).

To determine the effect of Ang-1 treatment on inflammatory activity in the lung, we first quantified granulocyte infiltration by measuring MPO activity in total lung homogenates and by counting neutrophils on BALf cytospin preparations. Compared to NVC, MPO activity and neutrophil numbers were markedly higher in lungs of HV_T_-ventilated mice (figures 6a and b). Administration of either 1 or 4 µg Ang-1 reduced granulocyte influx after HV_T_-ventilation (figure 6a). Supporting the MPO data, neutrophil numbers on BALf cytospin preparations were diminished in HV_T_-ventilated mice treated with 1 µg Ang-1 as compared to HV_T_-ventilated mice treated with vehicle (figure 6b). In addition, we stained lung sections for H&E to visualize granulocytes in pulmonary tissue. Figure 6c illustrates that both doses of Ang-1 prevented the appearance of granulocytes in lungs of HV_T_-ventilated mice, confirming the quantitative measures for infiltrating granulocytes.

### Effect of Ang-1 treatment on ventilator-induced chemokine and cytokine expression

**Figure 7:**
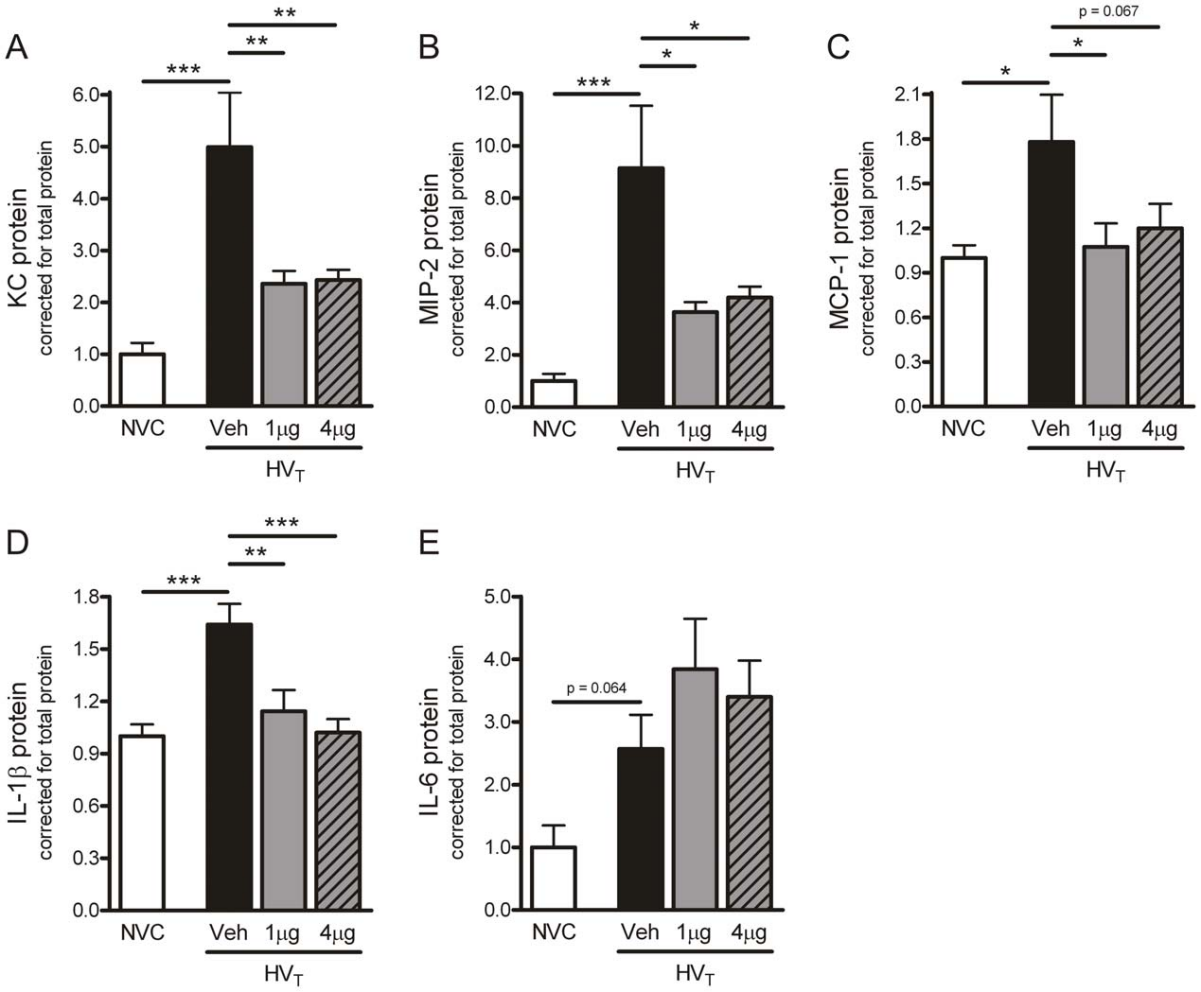
Angiopoietin (Ang)-1 treatment reduces chemokine and interleukin (IL)-1β protein expression. A–E: In total lung homogenates, protein expression of the chemo-attractants keratinocyte-derived chemokine (KC), macrophage inflammatory protein (MIP)-2, monocyte chemotactic protein (MCP)-1 and the pro-inflammatory cytokines IL-1β, IL-6 was determined by multiplex cytokine analysis. Data are depicted relative to NVC and expressed as mean ± SEM of 8–10 animals per group (* p<0.05, ** p<0.01, *** p<0.001). NVC  =  non-ventilated controls; HV_T_  =  mice ventilated with high tidal volumes; Veh, 1 µg, 4 µg  =  mice intravenously treated with either vehicle (sterile saline), Ang-1 (1 µg per animal), or Ang-1 (4 µg per animal).

The effect of Ang-1 treatment on the levels of inflammatory mediators expressed by pulmonary tissue during HV_T_-ventilation was determined as an additional measure of inflammatory activity in the lung. HV_T_-ventilation induced protein expression of the chemokines KC, MIP-2 and MCP-1 in comparison with NVC (figures 7a to c). Consistent with the diminished granulocyte influx, treatment with either 1 or 4 µg Ang-1 reduced the upregulation of these chemokines after 5 hours of HV_T_-ventilation. Moreover, higher levels of the pro-inflammatory cytokine IL-1β were found in pulmonary tissue of HV_T_-ventilated mice (figure 7d). The elevated protein expression of IL-6 did not reach statistical significance (p = 0.064, figure 7e). Although Ang-1 treatment decreased the expression of IL-1β protein compared to the HV_T_-vehicle group, it did not influence the expression of IL-6 (figures 7d and e). The anti-inflammatory cytokine IL-10 was below detection level in all experimental groups.

### Effect of Ang-1 treatment on ventilator-induced lung injury

**Figure 8:**
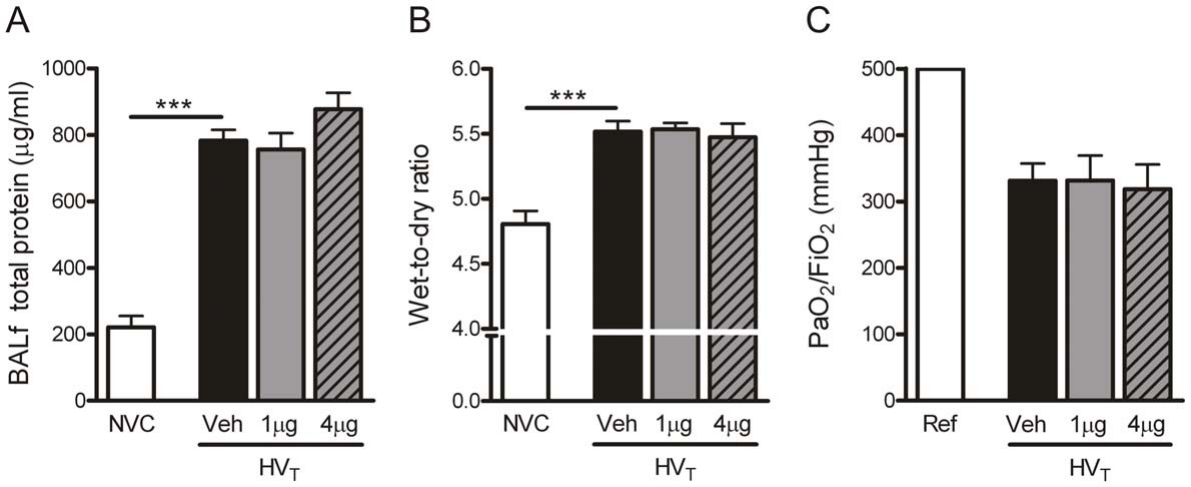
Angiopoietin (Ang)-1 treatment does not prevent alveolar-capillary permeability, pulmonary edema formation and impaired gas exchange. A: Alveolar-capillary permeability is represented by total protein levels in bronchoalveolar lavage fluid (BALf). B: Pulmonary edema is represented by wet-to-dry ratios of lung tissue. C: Oxygenation is represented by the ratio of arterial oxygen partial pressure to fractional inspired oxygen concentration (PaO_2_/FiO_2_). Data are expressed as mean ± SEM of 5-15 (A–B) or 14–18 (C) animals per group (*** p<0.001). Ref  =  reference bar (PaO_2_/FiO_2_ ratio for mice with non-injured lungs); NVC  =  non-ventilated controls; HV_T_  =  mice ventilated with high tidal volumes; Veh, 1 µg, 4 µg. =  mice intravenously treated with either vehicle (sterile saline), Ang-1 (1 µg per animal), or Ang-1 (4 µg per animal).

Ang-1 treatment suppressed the inflammatory activity in the lung. To determine the consequences for lung injury, we evaluated whether the increase in vascular leakage and decrease in oxygenation during HV_T_-ventilation could be restored by Ang-1 administration. As depicted in figures 8a and b, the increased BALf protein levels and pulmonary wet-to-dry ratios of HV_T_-ventilated mice were not affected by treatment with either 1 or 4 µg Ang-1. Furthermore, both doses of Ang-1 did not prevent the reduction in PaO_2_/FiO_2_ ratio caused by HV_T_-ventilation (figure 8c).

## Discussion

At the best of our knowledge this is the first report demonstrating that mechanical ventilation affects the Ang-Tie2 system in pulmonary tissue of healthy adult mice. Particularly in lungs of HV_T_-ventilated mice, we observed marked changes in the Ang-Tie2 system. Compared to NVC, 5 hours of HV_T_-ventilation resulted in downregulation of Ang-1, Ang-2 and Tie2 mRNA expression. In addition, Ang-1 and Ang-2 protein expression tended to decrease in HV_T_-ventilated mice at this time point. The major finding of the present study is that treatment with Ang-1 affected only specific aspects of VILI. Ang-1 administration at initiation of ventilation diminished granulocyte infiltration, as well as chemokine (KC, MIP-2, MCP-1), cytokine (IL-1β), VEGF and Ang-2 expression in lungs of HV_T_-ventilated mice. However, Ang-1 treatment did not prevent the increase in BALf protein level, the increase in pulmonary wet-to-dry ratio and the reduction in PaO_2_/FiO_2_ ratio induced by HV_T_-ventilation. This result was not only observed after administration of 1 µg of Ang-1 but also after administration of 4 µg of Ang-1. In preliminary experiments we also investigated whether a higher dose of Ang-1 (i.e. 8 µg per animal) would influence alveolar-capillary permeability and pulmonary edema formation induced by mechanical ventilation. However, 8 µg of Ang-1 appeared to have no effect on ventilator-induced vascular leakage as well. In view of these data, we would like to suggest that Ang-1 treatment does not prevent the aspects of VILI driven by mechanosensitive alterations in barrier properties [24] but will only regulate the pulmonary inflammation.

In experimental studies, mechanical ventilation has been described to induce destabilization of the alveolar-capillary barrier thereby leading to enhanced pulmonary permeability and edema formation [4]-[6]. Most models of VILI, however, applied very high inspiratory pressures or tidal volumes when compared to those used in the human setting [25]-[29]. To prevent shock and metabolic acidosis due to very high inspiratory pressures or tidal volumes, we used ventilation strategies with more clinically relevant ventilator settings [20]. Even in this relatively mild model of VILI, we observed that mechanical ventilation caused a modest, but significant increase in BALf protein level and pulmonary wet-to-dry ratio.

The importance of the Ang-Tie2 system has been appreciated in the development of vascular leakage and inflammation in pulmonary diseases like ALI/ARDS [11]. In this respect, Karmpaliotis et al. proposed that changes in the balance between VEGF (pro-leakage) and Ang-1 (anti-leakage) might contribute to vascular leakage in their murine model of lipopolysaccharide (LPS)-induced ALI [14]. Here we demonstrate that HV_T_-ventilation as such enhanced expression of VEGF protein, decreased expression of Ang-1, Ang-2 and Tie2 mRNA and tended to reduce expression of Ang-1 and Ang-2 protein compared to NVC. Since the mice were sacrificed after 5 hours of HV_T_-ventilation, we could not evaluate whether the downregulation in mRNA expression was followed by a significant decrease in protein expression at a later time point. Even so, the present study suggests that alterations in the Ang-Tie2 system are involved in the pathogenesis of VILI.

Kim et al. and Papapetropoulos et al. have demonstrated that Ang-1−mediated Tie2 signaling causes Akt phosphorylation thereby protecting the endothelial cells from apoptotic cell death [30], [31]. In our study, HV_T_-ventilation itself caused increased p-Akt levels compared to NVC which is in agreement with previous reports [32], [33]. Moreover, treatment with Ang-1 augmented p-Akt expression in lungs of HV_T_-ventilated mice. (Ang-1−mediated) Akt phosphorylation has been shown to inactivate the forkhead transcription factor FKHR1 subsequently preventing Ang-2 expression and destabilization of the endothelial barrier [34]. This observation supports our finding that HV_T_-ventilation as such reduced Ang-2 mRNA and that Ang-1 treatment downregulated the transcription of Ang-2 even further. The Ang-1−induced shift towards less Ang-2 production, thus reduced Tie2 antagonism, may protect against lung inflammation and injury during HV_T_-ventilation.

In agreement with previous reports [27]-[29], we observed that ventilator-induced lung injury was accompanied by enhanced pro-inflammation. In LPS-challenged animals, treatment with Ang-1 has already been shown to decrease leukocyte trafficking by reducing chemotactic, adhesive and pro-inflammatory mediators [17]. Our study is the first to show that Ang-1 administration downregulated infiltration of granulocytes and expression of the chemokines KC, MIP-2 and MCP-1 in an experimental model of VILI. Furthermore, we observed that administration of Ang-1 prevented the increase in the pro-inflammatory cytokine IL-1β in lungs of HV_T_-ventilated mice. Our data may suggest that the role of IL-1β might be less important in the development of vascular leakage during HV_T_- ventilation. Interestingly, IL-6 protein levels remained high in lungs of HV_T_-ventilated mice despite Ang-1 treatment. In pulmonary inflammation, IL-1β is primarily produced by activated alveolar macrophages whereas IL-6 may be derived from a wide variety of pulmonary cell types [35]. Since Tie2 is expressed on endothelial cells, neutrophils and macrophages [36], [37], our observations may imply that IL-6 is also derived from cells that do not express the Tie2 receptor and consequently do not respond to Ang-1. With respect to the clinical situation, it would be of interest to evaluate whether Ang-1 treatment would also be effective in attenuating lung inflammation in ventilated animals with pre-existing lung inflammation.

The angiogenic growth factor VEGF has been shown to increase capillary permeability and edema formation in various experimental models of pulmonary injury, including VILI [14], [38], [39]. Thurston et al. described that vascular leakage induced by VEGF may be counteracted by Ang-1 [40], [41]. In line with this notion, we observed that Ang-1 treatment completely abolished the increase in VEGF protein in lungs of HV_T_-ventilated mice. Nonetheless, it should be noted that Ang-1 treatment did not prevent alveolar-capillary permeability, pulmonary edema (i.e. vascular leakage) and impaired gas exchange induced by HV_T_-ventilation. These data are in apparent contrast with previously described protective effects of Ang-1 on vascular leakage in endotoxin-challenged animals [15]-[18] underlining that the pathways involved in endotoxin- and ventilator-induced lung injury are different. An explanation for this discrepancy might be that the enhanced inflammation is not the primary inducer of vascular leakage and impaired gas exchange during HV_T_-ventilation, as is the case in the induction of lung injury by LPS. It has been demonstrated that ventilator-induced mechanical stretch may also lead to the destabilization of alveolar-epithelial and capillary-endothelial barriers thereby resulting in increased vascular permeability and pulmonary edema [4]-[6]. Ang-1 administration will probably influence the capillary-endothelial but not the alveolar-epithelial barrier since the Tie2 receptor is mainly expressed on endothelial cells. Thus, the possibility remains that Ang-1 treatment is not capable of restoring lung injury induced by HV_T_-ventilation as it only modulates endothelial inflammation. The fact that Ang-1 prevents pulmonary vascular leakage in animals exposed to LPS, which induces a generalized inflammation primarily in the endothelial cells of the lung [42], supports this hypothesis.

Taken together, our data indicate that treatment with Ang-1 inhibits various aspects of VILI such as granulocyte infiltration, chemokine/cytokine and VEGF expression. However, Ang-1 treatment did not protect HV_T_-ventilated mice against the more crude parameters of VILI (i.e. vascular leakage and impaired gas exchange). In this respect, it is of interest that the TNF-α inhibitor Etanercept diminished inflammation and coagulation in the lungs of ventilated mice without influencing alveolar-capillary permeability and pulmonary edema, which is in line with our present results [43]. We propose that Ang-1 should not be applied to combat the mechanosensitive aspects of ventilator-induced lung injury in critically ill patients. Nonetheless, treatment with Ang-1 may well be considered as an anti-inflammatory therapy when inflammation is the primary inducer of lung injury, like in non-ventilated patients diagnosed with ALI/ARDS.
